# Perceiving a Resourcefulness: Longitudinal Study of the Sequential Mediation Model Linking Between Spiritual Leadership, Psychological Capital, Job Resources, and Work-to-Family Facilitation

**DOI:** 10.3389/fpsyg.2020.613360

**Published:** 2021-02-12

**Authors:** Pei Jiao, Changshien Lee

**Affiliations:** ^1^College of Marxism, Shandong University, Weihai, China; ^2^Department of Public Administration, Sookmyung Women’s University, Seoul, South Korea

**Keywords:** spiritual leadership, psychological capital, job resources, work-to-family facilitation, sequential mediation model

## Abstract

In order to improve our understanding of whether and how spiritual leadership promotes positive work-family outcomes from a resource perspective, this study proposed and tested for the first time a conceptual model incorporating job resources and psychological capital as the mediating factors between spiritual leadership and facilitation. We tested a theoretical model with date obtained from 529 Chinese workers who completed questionnaires in a four-wave survey. The results showed that the relationship between spiritual leadership and work-to-family facilitation was mediated by job resources alone, as well as job resources and psychological capital in sequence. Thus, this research may also pave the way for future spiritual leadership research on follower outcomes in other domains (e.g., community and school) by shifting the present spiritual leadership research focus from work outcomes to personal life. Implications for theory, managerial practices, limitation, and future research were discussed.

## Introduction

Historically, leadership research has tended to focus on the leader’s influence over outcomes such as organizational and individual effectiveness, performance, and success; thus overlooking work-family outcomes (Li et al., 2017). As the boundaries between work and private life become less distinct, it has become more imperative that modern organizations preserve and enhance employees’ health and well-being (Munn, 2013; Braun and Nieberle, 2017). To assume a more proactive role in assisting their employees in finding a balance between work and family needs (Li et al., 2017), many organizations have introduced a variety of family-friendly programs. These include flexible work schedules, on-site day-care centers, and parental leave to accommodate employees’ needs in that regard (Butts et al., 2013; Li et al., 2017). However, empirical studies have brought the efficacy of these programs into question, suggesting that a leader’s behavior in the workplace has a more significant effect on how employees manage work-family outcomes (Bagger and Li, 2014; Li et al., 2017). In recent years, researchers have begun to examine the influence of leader behavior on follower work-family outcomes (Kossek et al., 2011; Li et al., 2017).

Spiritual leadership may be an emerging perspective in which to extend theory in work-family research. Spiritual leadership incorporates vision, hope/faith, and altruistic love of a leader to intrinsically motivate the self and others to have a sense of spiritual awareness, such as spiritual well-being (Fry, 2003; Chen and Li, 2013). This leadership style focuses on satisfying employees’ spiritual needs, while also valuing employees’ perceptions of meaningfulness at work and in life (Fry, 2008). It also indicates a way that could inspire employees to work beyond role obligation, for the common good of the group and to treat the organizational stakeholders and customers conscientiously (Fry and Nisiewicz, 2013). Furthermore, considerable empirical evidence attests to the beneficial effects of spiritual leadership for employees and organizations. These benefits include life satisfaction and organizational commitment, organizational citizenship behavior, in-role job performance, and proactive behavior (Chen and Yang, 2012; Fry and Nisiewicz, 2013; Krishnakumar et al., 2014; Chen et al., 2019).

Despite these promising findings, work-family outcomes have been ignored in the existing spiritual leadership literature, partly because there are no frameworks to justify such a relationship.

In examining the process by which leaders impact follower outcomes, a growing body of work has highlighted the important role of resources (Breevaart et al., 2013). For example, researchers suggest that leaders can influence levels of resources for followers (e.g., Breevaart et al., 2013; Braun and Nieberle, 2017). Aligned with this argument, Yang et al. (2019) assert that employees, who perceive their leaders as exhibiting spiritual leadership behaviors such as vision, hope and faith, and altruistic love, are more likely to gain resources from these leaders. Furthermore, the resource-gain-development (RGD) theory (Wayne et al., 2007) proposes that the resources individuals attain from work may spill over to facilitate their performance in a family role. In addition, the Conservation of Resources (COR) theory (Hobfoll et al., 2018) suggests that resources do not exist individually but in caravans, for both individuals and organizations and thus personal resources emerge from shared environmental condition. From these theories, we would expect the model in which spiritual leadership as organization resources for followers would improve employee work-family facilitation directly and through fostering both job resources and personal resources [e.g., psychological capital (PsyCap)], thereby extending current views of spiritual leadership.

However, to the best of our knowledge, no research to date has been conducted focusing on understanding and empirically investigating whether spiritual leadership is related to employees’ work-family outcomes. Furthermore, research has not yet considered PsyCap and job resources as mechanisms through which perceived spiritual leadership is related to follower work-family outcomes. In addition, spiritual leadership theory is deeply rooted in Western culture (Chen et al., 2019), but Confucianism and spiritual leadership may share some commonalities. That is, compared with their individualistic counterparts, Chinese workers hold a more collectivist view (Chen et al., 2017). Hence, we may need to explore the applicability of the constructs mainly developed and applied in Western societies and organizations to other cultural contexts.

**FIGURE 1:**
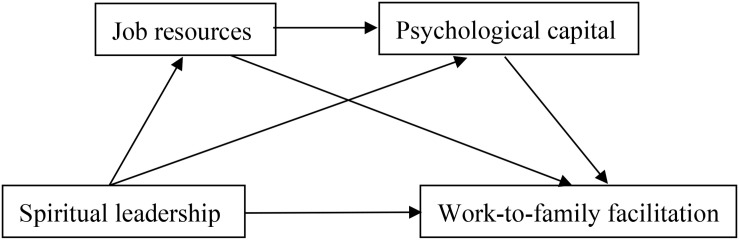
Hypothesized model depicting the potential mediating relationships.

To address the theoretical and practical research issues described above, our objective was to build on the COR theory (Hobfoll, 2002) and the RDG perspective (Wayne et al., 2007). We also intended to develop and test a serial multiple mediation model in which follower job resources and PsyCap acted as potential mediators in series between the perception of spiritual leadership and employee work-to-family facilitation (WFF) (see Figure 1). In addition, we used a four-wave design involving a Chinese sample in order to test the causal ordering of the model variables and to address the demand in the literature for longitudinal work-family studies (ten Brummelhuis and Bakker, 2012; Chen et al., 2017). This also enabled greater generalisability of findings across different contexts and countries (Chen et al., 2017).

## Theory and Hypotheses

### Spiritual Leadership

Spiritual leadership is an emerging paradigm used to examine leadership in terms of intrinsic motivation (Fry and Nisiewicz, 2013). Spiritual leadership is defined as “the values, attitudes, and behavior necessary to intrinsically motivate oneself and others so that they have a sense of spiritual well-being through calling and membership” (Fry et al., 2005, p. 836). This type of leadership aims to create vision and value congruence across the individual, team and organization levels and, ultimately, foster organizational commitment and productivity, thereby catering to individual well-being, corporate social responsibility, and organizational performance (Fry and Nisiewicz, 2013).

Conceptually, spiritual leadership comprises hope/faith, vision, and altruistic love (Fry, 2003). Vision refers to a compelling view of the future in which an individual or the organization wants to be (Fry, 2003). Altruistic love is “a sense of wholeness, harmony, and well-being produced through care, concern, and appreciation for both self and others” (Fry, 2003, p. 712). Hope is an expectation of fulfillment, while faith, which is the conviction that a thing unproved by physical evidence is true, adds certainty to hope (Fry, 2003). The process of spiritual leadership assumes that the interaction of vision, altruistic love, and hope/faith (i.e., intrinsic motivators) between leaders and followers leads to the achievement of their spiritual well-being (i.e., meaning/calling and membership). This, in turn, fosters higher levels of organizational commitment and productivity, satisfaction with life, sustainability, and financial performance (Fry and Nisiewicz, 2013; Hunsaker, 2016).

The above views suggest that leaders who model spiritual leadership values, communicate vision, cultivate followers’ hope and faith, and socially exchange altruistic love, are more likely to enhance their workers’ sense of spiritual well-being within the organization, which in turn can positively influence individual and organizational outcomes. We have established that spiritual leadership incorporates resources for followers such as vision, altruistic love, and hope/faith and determined that attitude and behavior in the work domain can be regarded as transferable resources and exported to the family domain (Greenhaus and Powell, 2006; Zhang et al., 2012). However, despite these employees’ individual and organizational benefits, literature on spiritual leadership has not addressed some compelling research issues until now. Does spiritual leadership enhance followers’ positive work-family outcomes? Do followers perceive sufficient psychological and work-related resourcefulness from spiritual leaders? Do these perceptions lead to positive work-family outcomes? Therefore, spiritual leadership constructs need more robust empirical investigation, hence, the current study addresses these issues.

### A Resource Perspective on WFF

WFF is defined as a form of synergy in which resources associated with work roles enhance or make participation in family roles easier (Voydanoff, 2004). One metanalysis suggested that work-related antecedents, such as leadership, tend to relate more strongly to WFF than family-to-work facilitation (Byron, 2005). Wayne et al. (2007) recently introduced the RGD perspective to illuminate how resources were related to WFF on a system level. They suggested that the key enablers of WFF were personal and environmental resources that contribute to the development of new skills and perspectives (developmental gains), positive emotion (affective gains), economic, social, or health assets (capital gains), and greater efficiency (efficiency gains) in a work system that enhances the functioning of the family system.

In particular, COR theory may provide insight into the dynamics of resource generation contributing to WFF. COR theory assumes that people are motivated to obtain and protect resources they value, but also that as resources are acquired, they tend to generate or invest them to gain other resources, which may result in positive outcomes such as better coping and well-being (Hobfoll, 2002). In line with this assumption, Xanthopoulou et al. (2007) suggested and found that job resources fostered personal resources, which in turn increased the levels of work engagement. In addition, according to Wayne et al. (2007), resources individuals attain from work may spill over to facilitate improved functioning in their family roles. Based on this theory, we address a process in which followers’ job resources, acquired from spiritual leaders, foster their personal resources, such as PsyCap, which in turn promote their WFF.

### Spiritual Leadership and WFF

As noted earlier, spiritual leadership could be a critical organizational resource for followers. Wayne et al. (2007) propose that when individuals obtain resources that can help them meet their goals in the work domain, they experience resource gains which are then applied to family domains. Thus, under the spiritual leadership context, employees, who perceive their leaders to be exhibiting spiritual leadership behaviors, are more likely to perceive a heightened level of resourcefulness from their leaders (Yang et al., 2019) that allows them to derive benefits from work and apply them to family life, thereby resulting in WFF.

Furthermore, according to Fry et al. (2005), spiritual leaders construct a vision that inspires employees to accomplish the organizational mission. The employees then are more likely to be encouraged to invest more time and effort into their work and organizations (Fry et al., 2005). By spending more time and effort on work, employees are able to gain a positive affect or learn skills and knowledge that can be transferred from the workplace and apply their family life to improve their family role performance (Wayne et al., 2007). In addition, spiritual leadership emphasizes altruistic love, which is the spiritual leader’s care and concern for others, to create an environment of joy, peace, and serenity (Chen and Li, 2013). Hence, when employees experience positive affect such as joy, peace, and serenity at work, they can transfer and apply these to the family, to their benefit. Taken together, we expect that perceived spiritual leadership would enhance followers’ WFF. To our knowledge, previous research has not yet tested this relationship. Thus, we propose Hypothesis 1: Perceptions of spiritual leadership relates positively to employee WFF.

### The Mediating Role of Job Resources

Job resources are defined as the aspects of a job that are functional in achieving work goals, reduce job demands, and stimulate personal growth and development (Bakker and Geurts, 2004). Although researchers offer various job resources (Bakker and Geurts, 2004; Voydanoff, 2004; Grzywacz and Butler, 2005; ten Brummelhuis and Bakker, 2012), we included four job resources that have been identified in previous studies as important factors that may enhance the experience of WFF. First, there is task significance, the degree to which the job has a substantial impact on the lives of other people (Hackman and Oldham, 1975). Second, there is autonomy, the degree to which employees are able to decide how to do their jobs (Voydanoff, 2004). Third, there are opportunities for development, the degree to which employees can develop themselves at work (Bakker and Geurts, 2004). Finally, there is performance feedback, the degree to which the job provides clear information about performance levels (Hackman and Oldham, 1975).

Researchers argue that, in general, the key responsibility of leaders is to provide resources for employees to successfully complete their work (Perry et al., 2010). In addition, Smircich and Morgan (1982) suggest that leaders define and shape followers’ immediate work environment. Aligned with this argument, we propose that spiritual leaders can foster followers’ job resources by shaping the work environment or providing resources. Spiritual leaders are more likely to shape jobs for followers to have more autonomy, feedback, task significance, and opportunities for development because spiritual leadership emphasizes the interaction of intrinsic motivators such as hope/faith, vision, and altruistic love to inspire followers to display their tenacity and pursue excellent performance (Wang et al., 2019). Leaders who exhibit altruistic love with coaching and teaching are more likely to delegate responsibilities, provide opportunities for workers to participate in work decision, and give them good feedback. Consequently, when leaders develop a compelling vision of the future for call for employees’ internal meaningfulness (Fry et al., 2005), employees may view their jobs as more significant (Fry et al., 2005). Leaders with hope/faith in a compelling vision may provide purpose to employees about their work roles and actively engage them above and beyond the formally defined responsibilities, encouraging them to take charge of their own development at work, thereby increasing their’ perceptions of opportunities to develop. In sum, we may expect that perceived spiritual leadership could foster employee job resources.

According to Wayne et al. (2007), the follower job resources facilitated by leaders may lead to their WFF. They argue that it is possible to predict WFF from jobs that are psychologically enriched. Voydanoff (2004) found that within-domain work resources such as autonomy, meaningful work, and learning opportunities are positively related to WFF. Further, Bakker and Geurts (2004) found that job resources such as autonomy, possibilities for development, and performance feedback translate into a positive work-family interference. Taken together, we may expect that spiritual leadership fosters followers’ perceptions of job resources, leading to promotion of their WFF. However, no known studies have examined the mediating role of job resources in the relationship between spiritual leadership and WFF. To this end, we offer Hypothesis 2: Job resources mediate the relationship between perceived spiritual leadership and WFF.

### The Mediating Role of PsyCap

PsyCap refers to “an individual’s positive psychological state of development.” This is characterized by: “(1) having confidence (efficacy) to take on and put in the necessary effort to succeed at challenging tasks; (2) making a positive attribution (optimism) about succeeding now and in the future; (3) persevering toward goals and, when necessary, redirecting paths to goals (hope) in order to succeed; and (4) when beset by problems and adversity, sustaining and bouncing back and even beyond (resilience) to attain success” (Luthans et al., 2007, p. 542).

Regarding the mediating role of PsyCap, Zhang et al. (2012) addressed the impact of leadership on work-family consequences by focusing on the mediating roles of positive psychological states. Aligned with the above, we propose that the positive relationship between perceived spiritual leadership and followers’ WFF is associated with followers’ PsyCap that spiritual leaders nurture.

To establish the mediating role of followers’ PsyCap in the relationship between perceived spiritual leadership and their WFF, two links must be established. First, spiritual leadership must be related to followers’ PsyCap. When a leader develops a vision of a long-term challenging, desirable, compelling, and different future with altruistic values and shares it with their employees (Fry et al., 2005), employees are more likely to facilitate their ability to set goals and believe that those goals can be achieved and create belief in one’s ability to successfully (Gooty et al., 2009), thereby enhancing their self-efficacy. Chen et al. (2012) found a positive effect of spiritual leadership on employees’ self-efficacy perceptions. Furthermore, spiritual leaders cultivate followers’ hope by articulating a clear and sufficiently challenging goal to motivate them (Chen et al., 2019), which is vital in creating targets toward which people can direct their agency (Luthans et al., 2007). In addition, spiritual leaders foster employee optimism by creating a desirable, compelling vision for followers to portray a picture for followers to evaluate current and future circumstances favorably (Wang et al., 2019). Finally, this type of leader nurtures followers’ resilience by actively encouraging followers to take calculated risks, and to seek opportunities and solve complex organizational problems (Chen and Li, 2013). In sum, we would expect that perceived spiritual leadership may facilitate employees’ PsyCap of self-efficacy, optimism, hope, and resilience.

The second link that must be established is that followers’ PsyCap must be related to their WFF. Wayne et al. (2007) propose that personal characteristics (e.g., self-efficacy) enable WFF because they cause the individual to more readily experience positive emotional states, seek positive developmental experiences, and earn status and other assets. Choi et al. (2018) found a positive effect of PsyCap on work-to-family enrichment, a construct related to facilitation. Moreover, a previous study showed that PsyCap acts as a mediator in the relationship between spiritual leadership and employee performance (Baykal and Zehir, 2018). This finding may provide insight into the mediating role of PsyCap in the spiritual leadership–WFF relationship. Given the positive relationship between job performance and parental performance (Friedman and Greenhaus, 2000), we may logically expect spiritual leadership to foster employees’ PsyCap, and this in turn promotes their WFF. However, to date, no research has empirically examined this relationship. To this end, we offer Hypothesis 3: Employees’ PsyCap mediates the relationship between perceived spiritual leadership and their WFF.

### The Sequential Mediating Roles of Job Resources and PsyCap

As Breevaart et al. (2013) indicated that leaders may encourage followers to actively increase their own resources, spiritual leaders also would facilitate employees’ job resources, as discussed in Hypothesis 2. According to COR theory (Hobfoll, 2002), we argue that job resources that are obtained from spiritual leaders can foster personal resources such as PsyCap, which in turn promote WFF. In support of this notion, Xanthopoulou et al. (2007), based on the COR theory, suggested and revealed that job resources (e.g., autonomy and opportunities for professional development) were positively related to personal resources (e.g., self-efficacy and optimism). Moreover, ten Brummelhuis and Bakker (2012), also based on the COR theory, proposed that job resources strengthen personal resources. In a similar vein, Luthans et al. (2006) showed that a resourceful work environment activates employees’ PsyCap. In short, followers’ job resources that are obtained from their leaders can foster their PsyCap. Additionally, as addressed in Hypothesis 3, we suggest PsyCap is related to WFF. Hence, we propose a two-stage mediation process, in which the perceptions of spiritual leadership nurture followers’ job resources, which subsequently strengthen their PsyCap. Ultimately, this leads to the promotion of their WFF. Thus, we offer Hypothesis 4: Perceived spiritual leadership positively relates indirectly to employees’ WFF through their job resources, and consequently through PsyCap.

## Materials and Methods

### Participants and Procedures

We collected data from 529 full-time front-line employees working in a large pharmaceutical company located in Jiangsu province, China. Participants were workers from different functional departments, such as production, research and development, customer service, marketing and sales, finance and accounting, and human resources, etc. With the assistance of the human resource manager, an original sample of 1,100 full-time employees was randomly drawn from the membership list of the firm (*N* = 4,400). After obtaining permission from the departments’ leaders, we administered the questionnaires in four waves, with approximately 3 months separating each survey wave. The 3-month time lag between consecutive waves was chosen (1) to test the causal ordering of the model variables, (2) allow us to mitigate problems of reverse causation, (3) reduce common method variance (CMV) (Podsakoff et al., 2003), and (4) provide sufficient separation between our measures, while not spacing surveys so far apart that it would increase participant attrition (Siu et al., 2015). The research team gave all participants their survey packets containing copies of our questionnaire, cover letters, and return envelopes during work hours. They then asked the participants to personally complete and return their questionnaires directly to one of the researchers using a prepaid return envelope in a week. We also provided a high-quality pen as a gift to each participant to show our gratitude and used a series of numbers (Zhang et al., 2012) rather than participant names to identify each questionnaire to guarantee anonymity. We assured respondents through the cover letter that we would keep their responses completely confidential; that their participation was voluntary; and that the objective of the survey was to evaluate the effectiveness of spiritual leadership in terms of work-family outcomes.

We assessed the predictor of spiritual leadership and control variables at time 1 (T1), one mediating variable of job resources at time 2 (T2), another mediating variable of PsyCap at time 3 (T3), and the criterion variable of WFF and the marker variable of formalization at time 4 (T4). After matching the T1, T2, T3, and T4 surveys three times in sequence using a matching code, we achieved a 4-wave matched sample of 630 (57.27%) full-time workers. After excluding respondents who were careless responding (i.e., large number of blanks) during the data entry process, we obtained 529 complete and useable responses. This final sample had more males (58.4%) than females (41.6%). Respondent ages were group as 25 years and younger (13.4%), 26–35 (50. 7%), 36–45 (28.9%), 46–55 (6.6%), and 56 and older (4%). Approximately two-thirds (70.4%) of the respondents had obtained a bachelor’s or associate degree. The organizational tenure of the employees sampled was less than 2 years (6.4%), 2–3 (15.5%), 4–8 (24.6%), 9–13 (22.3%), and more than 13 years (31.3%).

### Analytical Approach

The data was analyzed using two-step approach involving confirmatory factor analysis (CFA) and structural equation modeling (SEM). Both the CFA measurement models and the structural models were estimated using the Mplus 7.4 program with maximum likelihood estimation (Muthén and Muthén, 1998-2012). Prior to examining the hypotheses, we estimated a series of nested measurement models to assess the discriminant validity of our substantive constructs. These analyses were conducted using items as indicators of the latent variables. Next, the severity of CMV was examined with the Comprehensive CFA Marker Technique approach (Williams et al., 2010). This analysis was performed using item parcels as indicators because the marker variable should not load on too many indicators (i.e., 53 items in this study), resulting in the serious inflation of measurement errors.

To test the hypotheses, we then assessed competing structural models with the model comparison strategy, through which the best model was identified. Since multiple items within a single latent variable cause the inflation of measurement errors, we developed parcels by grouping items within each scale to serve as indicators of the latent variable when the number of items for the variable exceeded nine. Thus, we randomly created four parcels for spiritual leadership and three parcels for PsyCap and job resources. We used the mean score of the items constituting each parcel as the score for the corresponding indicator (Jiang and Jiang, 2015). Based on a two-index strategy by Hu and Bentler (1999), well-fitting models were defined as those that had a standardized root-mean-square residual (SRMR) ≤ 0.08 and met at least one of the following criteria: root-mean-square error of approximation (RMSEA) ≤ 0.06 or comparative fit index (CFI) ≥ 0.95. We tested the significance of indirect effects using a bootstrapping procedure with 5,000 samples in the Mplus 7.4 program (Muthén, 2011). The five control variables of age, gender, education level, organizational tenure, and ethical leadership, were allowed to relate with all model variables.

### Measurement

As the surveys were initially written in English, following a standard translation and back-translation procedures by Brislin (1980), we translated the English survey into Chinese and then an English professor of the first author’s university conducted back-translation from Chinese to English. For all measurement instruments, we adopted a five-point Likert-type scale questionnaire ranging from (1 “strongly disagree”) to (5 “strongly agree”).

### Spiritual Leadership

We assessed spiritual leadership at T1 using 14 of the 17 items of the Spiritual Leadership Scale (Fry et al., 2005). We first administered the 17 items reflecting three dimensions of vision (five items), hope/faith (five items), and altruistic love (seven items). However, CFA of this scale revealed that the three-factor model fit the data poorly [χ^2^(119) = 1166.46, *p* < 0.001; CFI = 0.840, RMSEA = 0.129, and SRMR = 0.089]. Three items (one regarding hope and two regarding altruistic love) exhibited very low factor loadings (−0.01, −0.09, and −0.01) and highly correlated error terms, so these items were dropped. Subsequently, a well-fitted first-order model of three factors with 14 items emerged [χ^2^(74) = 262.46, *p* < 0.001; CFI = 0.968, RMSEA = 0.069, and SRMR = 0.028]. Furthermore, a second-order factor model produced the same fit to the data as the first-order model where the three dimensions loaded on a higher spiritual factor. In addition, the second-order and first-order models exhibited a better fit than the single factor model, in which all 14 items loaded on a single factor [χ^2^(77) = 504.08, *p* < 0.001; CFI = 0.928, RMSEA = 0.102, and SRMR = 0.038]. Overrall, the 14-item spiritual leadership scale demonstrated a high internal reliability of α = 0.95. Based on these results, we created a higher-order composite factor to operationalize spiritual leadership.

### PsyCap

We measured PsyCap at T3 using the shorter 12-item version of the Psychological Capital Questionnaire (PCQ) developed by Luthans et al. (2007). The PCQ-12 includes three items to measure efficacy, four items to measure hope, three to measure resilience, and two to measure optimism. This measure has demonstrated acceptable reliability and discriminant validity in previous research (e.g., Avey et al., 2011). The overall 12-item scale demonstrated high internal reliability with α > 0.92.

### Job Resources

We assessed job resources at T2 using the four subscales of task significance, job autonomy, performance feedback, and opportunities for development. Task significance and performance feedback were measured using three items from the Job Diagnostic Survey (JDS; Hackman and Oldham, 1975). We then measured job autonomy and opportunities for development on a three-item scale developed by Voydanoff (2004). We used these four subscales to form a second-order composite factor, subsequently termed job resources. The CFA of the four second-order model confirmed the four-factor structure [χ^2^(50) = 193.22, *p* < 0.001, CFI = 0.960, RMSEA = 0.074, and SRMR = 0.036]. In addition, correlation analyses indicated that these three subscales had relatively high correlations, ranging from 0.58 to 0.74. The job resources scale demonstrated high internal reliability of α = 0.91. Therefore, job resources can be considered a core construct.

### WFF

We measured WFF at T4 with four items taken from Van Steenbergen et al.’s (2007) WFF scale. We selected items that were highly loaded from 0.83 or more to 0.88 in the explanatory factor analysis (EFA) (see Appendix of Van Steenbergen et al., 2007). Cronbach’s alpha for WFF was 0.93, indicating high internal consistency in our sample. A sample item of WFF was, “The skills I use at work help me to better handle matters at home.”

### Control Variables

We controlled the demographic variables of gender, age, organizational tenure, and education level of the respondents in this study because these variables have been influential in work-family processes (e.g., Grzywacz and Butler, 2005; McNall et al., 2015). We also controlled for perceptions of ethical leadership because, as Reave (2005) suggested, spiritual leadership closely aligns with ethical leadership and requires moral character and an ethical climate. We measured leaders’ ethical leadership using 5 items from the Ethical Leadership Questionnaire with a 10-item scale developed by Brown et al. (2005) at T1. An example item was “My leader conducts his/her personal life in an ethical manner.” Cronbach’s alpha for the scale was 0.78.

### Formalization

This construct acted as a marker variable in the current study and was assessed at T4 with three items developed by Pugh et al. (1968). This scale had a Cronbach alpha of 0.75. An example of an item in this scale was, “The organization has a large number of written rules and policies.”

## Results

### Descriptive Statistics and Correlations

Table 1 presents the means, standard deviations, and zero-order correlations of all variables. As shown, all mediation variables were significantly correlated in the hypothesized mediation direction. Formalization was selected as the best estimate of CMV in a data set (Williams et al., 2010) as it displayed very small relationships with the substantive variables (−0.04 to 0.10).

**TABLE 1:** Means, standard deviations, and zero-order correlations among study variables.

	***M***	***SD***	**1**	**2**	**3**	**4**	**5**	**6**	**7**	**8**	**9**	**10**
1. Age	2.32	0.81	−									
2. Gender	1.42	0.49	–0.03	−								
3. Education	2.20	0.88	−0.16**	−0.19**	−							
4. Tenure	3.40	1.22	0.66**	–0.02	–0.003	−						
5. SPL	3.48	0.86	0.08	–0.03	–0.05	0.01	(0.95)					
6. JR	3.36	0.72	0.07	–0.04	–0.02	0.06	0.75**	(0.91)				
7. PsyCap	3.46	0.69	0.05	–0.03	–0.06	0.05	0.61**	0.77**	(0.92)			
8. WFF	3.60	0.85	0.04	–0.05	–0.05	0.04	0.62**	0.69**	63**	(0.93)		
9. EL	2.97	0.81	0.01	0.07	0.07	–0.02	0.11**	0.07	0.04	08	(0.78)	
10. FO	2.76	0.92	0.10*	0.07	−0.14**	0.04	0.06	0.07	0.10**	0.04	–0.04	(0.75)

Age: 1 = “25 years and younger,” 2 = “26 to 35 years,” 3 = “36 to 45 years,” 4 = “46 to 55 years,” 5 = “56 and older.” Gender: 1 = “male,” 2 = “female.” Education: 1 = “high school and below,” 2 = “associate degree,” 3 = “bachelor degree,” 4 = “master degree,” 5 = “doctor degree.” Tenure: 1 = “below 2 years,” 2 = “2 to 3 years,” 3 = “4 to 8 years,” 4 = “9 to 13 years,” 5 = “14 years and above.” SPL, spiritual leadership; JR, job resources; PsyCap, psychological capital; EL, ethical leadership; FO, formalization; WFF, work-to-family facilitation. Reliabilities (coefficient alpha) appear in parentheses on the diagonal. *p < 0.05, **p < 0.01.

### Measurement Model

To examine the discriminant validity of our substantive constructs (i.e., spiritual and ethical leadership, job resources, PsyCap, and WFF), we conducted CFA. As shown in Table 2, the proposed five-factor Model demonstrated adequate fit with the data [χ^2^ (1070) = 2394.03, *p* < 0.001; CFI = 0.92, RMSEA = 0.048, SRMR = 0.039]. Against this five-factor Model, we assessed four alternative models that reduced the number of factors by combining some of the five factors into one factor (Wang and Hsieh, 2014). As Table 2 shows, the five-factor Model fit the data considerably better compared with alternative models. Chi-square difference tests also demonstrated that the 5-factor Model fit the data best. Hence, we could confirm the discriminant validity of the five variables.

**TABLE 2:** Comparison of measurement models.

**Model**	**χ ^2^**	**df**	**△χ ^2^**	**CFI**	**RMSEA**	**SRMR**
5-factor model	2394.03	1070		0.920	0.048	0.039
4-factor model	3044.19	1074	650.06***	0.881	0.059	0.047
3-factor model	3735.55	1077	1641.36***	0.840	0.068	0.061
2-factor model	4377.73	1079	1983.54***	0.801	0.076	0.064
1-factor model	6007.31	1080	3613.14***	0.703	0.093	0.079

5-factor: all five study variables served as independent factors; 4-factor: psychological capital and job resources loaded on one factor; 3-factor: psychological capital, job resources, and ethical leadership loaded on one factor; 2-factor: psychological capital, job resources, ethical leadership, and work-to- family facilitation loaded on one factor; 1-factor: all variables loaded on one factor. ***p < 0.001.

### Common Method Variance

To test CMV using the Comprehensive CFA Marker Technique approach (Williams et al., 2010), we first estimated the CFA Model, which provides a complete set of correlations among the five substantive constructs and the marker variable. Next, we evaluated the Baseline Model, which allows the five substantive factors to be correlated with each other, but has an orthogonal marker latent variable with its indicators fixed at three factor loadings (i.e., 0.741, 0.774, 0.874) and three error variances (i.e., 0.604, 0.734, 0.501) that were obtained from the CFA Model (Williams et al., 2010). Against this Baseline Model, we investigated two alternative models. The Method-C Model is identical to the Baseline Model, except that the 21 factor loadings from the marker latent variable to the substantive indicators must be equivalent. The Method-U Model is similar to the Method-C Model, except that the 21 factor loadings from the marker latent variable to the substantive indicators can have different estimates (Williams et al., 2010). All models provided a good fit to the data, as shown in Table 3.

**TABLE 3:** CFA results for common method variance test.

**Model**	**X^2^**	**df**	**△X^2^**	**CFI**	**RMSEA**	**SRMR**
CFA Model	317.84	194		0.986	0.035	0.024
Baseline Model	323.98	205		0.986	0.033	0.032
Method-C Model	323.41	205	^a^19.68	0.985	0.034	0.030
Method-U Model	303.73	186	^b^20.25	0.986	0.035	0.023

*CFA Model: A basic CFA model with a complete set of correlations among the studied constructs and the marker variable. Baseline Model: CFA Model with an orthogonal marker variable with its indicators’ fixed factor loadings and error variances obtained from CFA Model. Method-C Model: Baseline Model with loadings from the method factor to substantive indicators constrained to be equal. Method-U Model: Baseline Model with unequal loadings from the marker factor to substantive indicators.*

*^*a*^19.68: X^2^ difference between Method-C Model and Method-U Model;*

*^*b*^20.25: X^2^ difference between Baseline Model and Method-U Model.*

A comparison of the Baseline Model to the Method-C Model and the Method-U Model and of the Method-U Model to the Method-C Model provides a test of the presence and equality of method effects associated with the marker latent variable (see Williams et al., 2010, p. 493–494), respectively. As Table 3 shows, under the parsimony of model principle, the results of the χ^2^ difference test suggested that the Method-C Model did not fit significantly better than the Baseline Model and the Method-U Model, thereby no evidence of the presence of CMV and equal CMV was found in the data. Taken together, the CMV was not significant enough to proceed with hypothesis testing.

### Hypotheses Testing

**FIGURE 2:**
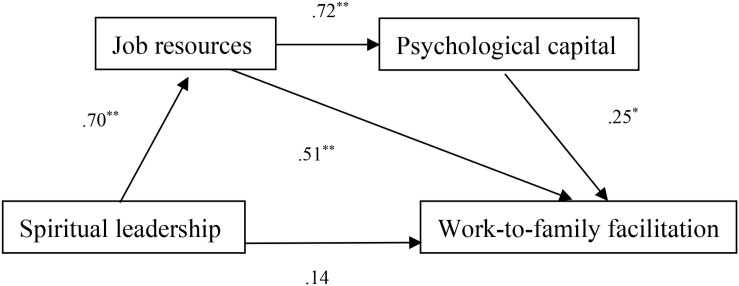
The final structural model with unstandardized path coefficients. *p < 0.05. **p < 0.001.

Table 4 presents the fit statistics for the hypothesized (Model 1) and alternative models. The hypothesized model fit the data well [χ^2^(198) = 299.15, *p* < 0.001; CFI = 0.988, RMSEA = 0.031, and SRMR = 0.023]. In order to identify the best-fitting model, six other theoretically plausible alternative models (Model 2–Model 7) were compared with the hypothesized one, considering the role of job resources and PsyCap as mediators. In Model 2, we deleted a direct path from spiritual leadership to PsyCap from the hypothesized model. In Model 3, the link between job resources and WFF was removed. In Model 4, the link between spiritual leadership and WFF was deleted. Model 5 was identical to Model 1, except for the deletion of two direct paths from spiritual leadership to both PsyCap and WFF. In Model 6, both the link between spiritual leadership and WFF and that between job resources and WFF were removed. In our last alternative model, Model 7, we removed two direct paths from spiritual leadership to PsyCap and WFF and one direct path from job resources to WFF. As shown in Table 4, under the rule of parsimony, the results of the chi-square difference test suggested that Model 2 best fitted our data. In conclusion, the Model 2 was the best representation of the data and was used to analyze the hypothesized relationships (see Figure 2).

**TABLE 4:** Fit indices of the hypothesized and alternative models.

**Model**	**X^2^**	**df**	**△X^2^**	**CFI**	**RMSEA**	**SRMR**
Model 1	299.15	198		0.988	0.031	0.023
Model 2	300.47	199	0.132	0.988	0.031	0.023
Model 3	320.77	199	21.62	0.985	0.034	0.026
Model 4	303.57	199	4.42	0.987	0.032	0.024
Model 5	305.58	200	6.43	0.987	0.032	0.024
Model 6	371.28	200	72.13	0.979	0.040	0.043
Model 7	371.81	201	72.66	0.979	0.040	0.042

*Model 1: the hypothesized model; Model 2: Model 1 – path spiritual leadership (SP)→PsyCap(PC); Model 3: Model 1 – path job resources (JB)→work-to-family facilitation (WFF); Model 4: Model 1 – SP→WFF; Model 5: Model 1 – (path SP→WFF + path SP→PC); Model 6 – (path SP→WFF + path JB→WFF); Model 7 – (path SP→WFF + path JB→WFF + path SP→PC).*

As shown in Table 5, there was a significant total effect of spiritual leadership on WFF: *B* = 0.62, *p* < 0.001. However, when the variance associated with the mediators was controlled, the direct effect of spiritual leadership on WFF was considerably reduced, and also not significant: *B* = 0.14, *p* = 0.058. Thus, the findings did not support Hypothesis 1. None of the covariates had significant effects on the WFF.

**TABLE 5:** Direct and indirect effects for the final model.

**Direct effect**				**Indirect effect**			
**Model path**	**Effect**	***SE***	***P***	**Model path**	**Effect**	**SE**	**95% CI**
SPL→JB	0.70	0.04	0.000	SPL(T1)→JB(T2)→WFF(T4)	0.36	0.09	0.18–0.54
JB →PsyCap	0.72	0.04	0.000	SPL(T1)→JB(T2)→PsyCap(T3)→WFF(T4)	0.12	0.06	0.02–0.24
SPL→WFF	0.14	0.07	0.058				
JB→WFF	0.51	0.12	0.000				
PsyCap→WFF	0.25	0.11	0.025				
Total direct effect	0.62	0.04	0.000	Total indirect effect	0.48	0.07	0.36–0.62

*Unstandardized coefficients are reported. SPL, spiritual leadership; PsyCap, psychological capital; JB, job resources; WFF, work-to-family facilitation. The control variables are not shown in this table for the sake of parsimony.*

As Table 5 shows, regarding the indirect effects, the bootstrap estimation showed that the total indirect effect of spiritual leadership on WFF was significant: estimate = 0.48, 95% CI (0.36–0.62). Next, we delineated the mediated effect into three components. First, job resources were mediators in the spiritual leadership–WFF association [estimate = 0.36, 95% CI (0.18–0.54)], supporting Hypothesis 2. In other words, perceptions of spiritual leadership at T1 uniquely fostered followers’ job resources at T2 (*B* = 0.70, *p* < 0.001), which consequently contributed to experiencing their WFF (*B* = 0.51, *p* < 0.001). In contrast, PsyCap did not significantly mediate the relationship between spiritual leadership and WFF, which did not support Hypothesis 3. Finally, we examined whether job resources and PsyCap would operate in sequence to mediate the relationship between spiritual leadership and WFF. The results showed that perceptions of spiritual leadership at T1 can facilitate followers’ job resources at T2 (*B* = 0.70, *p* < 0.001), and that these resources result in strengthening their PsyCap at T3 (*B* = 0.72, *p* < 0.001). This in turn spills over to the family domain and improves family role performance at T4 (*B* = 0.25, *p* < 0.05). Overall, the three-path mediation was significant: estimate = 0.12, 95% CI (0.02–0.24). Accordingly, the findings supported Hypothesis 4.

## Discussion

Using the RGD perspective and COR theory as the theoretical underpinnings, the current study developed the serial mediation model. It included the direct effect between spiritual leadership and follower WFF, as well as all possible indirect effects in this relationship. The relationships given above were tested based on field data from a four-wave survey in China. The results partly supported our model, but also suggested differential relations between the variables of interest.

Unexpectedly, spiritual leadership did not exert a significant positive effect on followers’ WFF. This result suggests that the scope of spiritual leadership may not predict a proximal variable such as WFF (Boyar and Mosley, 2007). WFF depends on an individual’s perception of skills and abilities that enhance role completion (Boyar and Mosley, 2007). Indeed, in this study, employees’ job resources and volatile resources of PsyCap were significantly related to facilitation. Thus, our finding may provide insight into the underlying mechanism or process causing WFF, suggesting expending the RGD model when examining work-family outcomes in future studies.

Consistent with the proposed theoretical model, job resources alone, as well as job resources and PsyCap in sequence, mediated the relationship between spiritual leadership and follower WFF. These results contribute significantly in explaining the underlying mechanisms of how spiritual leaders effectively improve their followers’ WFF. These results also align with COR theory such that when employees receive sufficient resources from spiritual leaders to increase their job resources, they invest these resources to activate their PsyCap, which may result in a positive work-family interface. In contrast to job resources, PsyCap did not serve as a mediating mechanism in the relationship between spiritual leadership and follower WFF. This is surprising. The results may be attributed to the dark side (negative effects or costs) of spiritual leadership, coupled with the nature of Chinese cultural work characteristics, job resources, and PsyCap included in this study.

Krishnakumar et al. (2014) assert that when individuals choose spirituality at the expense of rationality, they become vulnerable to manipulation, and thus spiritual leaders may attempt to coerce employees into overworking by placing the needs of the organization above their own needs. Furthermore, due to the collectivist view of work, Chinese workers tend to place more emphasis on work and see work as contributing to the family rather than competing with it (Spector, 2007). Thus, in the context of spiritual leadership, they are more likely to blindly devote themselves to an organization. In addition, job resources are traditionally seen as instrumental for employees to fulfill their work tasks, but PsyCap, as personal resources, operates mainly at an affective-cognitive level and less at a behavioral-practical level (Xanthopoulou et al., 2007). Therefore, we argue that, in the Chinese context, spiritual leaders are more likely to attempt to manipulate employees into overworking, wherein leaders and employees pay far more attention to job resources rather than their PsyCap. Consequently, we may conclude that in the Chinese context, consistent with our findings, job resources play a mediating role in the spiritual leadership-follower WFF relationship, whereas PsyCap does not.

Another explanation is that, given that the four factors of PsyCap have not only a common but also distinct properties (Luthans et al., 2007), only the unique component of PsyCap might explain the relationship between spiritual leadership and WFF. In our *post hoc* analysis, self-efficacy [estimate = 0.021, 95% CI (0.002–0.051)], a key component of PsyCap, was found to significantly mediate the relationship between WFF, but other components such as optimism [estimate = 0.017, 95% CI (−0.003 to 0.045)], hope [estimate = 0.007, 95% CI (−0.009 to 0.028]), and resilience [estimate = 0.011, 95% CI (−0.005 to 0.034) did not. Consistent with this result, Chen and Li (2013) suggest that spiritual leaders seek to affect the self-efficacy of their subordinates, so that they would internalize the values and vision of the organization, which would then reflect in their own system of values. In their research, they demonstrated that self-efficacy mediated the relationship between spiritual leadership and employee outcomes.

### Theoretical Implications

The results of this study provide several important contributions to the literature on leadership, positive psychological states, job resources, and work-family outcomes.

First, the evidence that spiritual leadership effectively promotes employees’ WFF through fostering their job resources may shift the previous spiritual leadership research concern from work outcomes to personal life, enabling the two research areas of spiritual leadership and work-family interface to converge. The convergence of these two research areas can direct researchers to explore the interface between spiritual leadership and other followers’ work-family outcomes, including family undermining, work-family conflict, and work-life balance (Zhang et al., 2012). In addition, we have also responded to the call from Chen and Li (2013) to study more spiritual leadership outcomes.

Second, previous studies on spiritual leadership have primarily focused on calling and membership as mediators to account for how spiritual leadership influences employee outcomes (Chen and Yang, 2012; Chen and Li, 2013). However, from a resource perspective, this study uncovered the two sequential mediating mechanisms (i.e., job resources and PsyCap) in the association of spiritual leadership with WFF. Thus, our study not only enriches the spiritual leadership and work family literature, but also echoes the claim to integrate spiritual leadership with other relevant theoretical frameworks (Chen et al., 2019) and to examine additional followers’ mediating mechanisms in spiritual leadership research (Chen and Li, 2013).

Finally, as noted earlier, spiritual leadership theory is deeply rooted in Western culture (Chen et al., 2019), and most of the research exploring work-family relationships has largely examined workers in Western countries (Shek, 2006). Compared with their individualistic counterparts in Western culture, Chinese workers hold a more collectivist and Confucian view. Thus, findings from previous researches performed in the West may not be applicable to workers in the East (Chen et al., 2017). However, by examining workers in China, our findings may expand the external validity of previous research examining workers from Western, North American societies. Furthermore, our results may improve the internal validity of previous authors’ findings using cross-sectional studies, since we collected data using a four-wave design to mitigate problems of reverse causation and CMV, and controlled for ethical leadership as an alternative explanation to obtain stronger conclusions.

### Limitations and Future Research

The first limitation is that observations were based solely on self-reports, which could be subject to self-enhancement bias. In separating the predictors, mediators, and outcomes in time from each other, the four-wave longitudinal design at least partly reduced the risk of common method bias (Podsakoff et al., 2003). However, future studies would benefit from using various sources of data, such as peer or supervisor ratings.

Second, we obtained the data used in this study from a single organization, thereby limiting the generalizability of the results. Conducting this study within a single organization where organizational culture is consistent and employees share common values provided the advantage of controlling for potential organization-level confounding variables (Yang et al., 2019). However, future research should replicate this model in multiple organizational settings in order to increase the generalizability of the findings (Yang et al., 2019).

Third, we did not examine the effects of the dimensions of spiritual leadership on employee WFF further. Spiritual leadership includes dimensions of vision, hope/faith, and altruistic love (Fry, 2003). Previous studies have demonstrated that leaders’ altruistic love and vision enhance spiritual well-being in organizations (Anderson and Sun, 2017; Chen et al., 2019). Specifically, in this study, although PsyCap did not mediate the spiritual leadership–WFF relationship, in the *post hoc* analysis, we observed that self-efficacy significantly mediated the relationship between spiritual leadership and WFF. Such findings may raise various questions for future research as follows: First, which factors of spiritual leadership constructs are most predictive of employee work-family outcomes? Second, if different components of spiritual leadership have different effects on employee work-family interface, which situation factors are more likely to influence these effects? Third, which facet of PsyCap and job resources constructs are the most powerful mechanism through which spiritual leadership influences employee work-family outcomes? Focussing on the dimensional level of spiritual leadership, PsyCap, job resources, and employee work-family outcomes may allow researchers to obtain deeper and richer insights about their relationship than what is available at the construct level (Li et al., 2017).

Fourth, although this study examined two potential mediating mechanisms, PsyCap and job resources, to build a complete picture of how leadership behaviors can drive WFF, future research could explore other mechanisms that contribute to the relationship between spiritual leadership and employee WFF. For example, as researchers noted, other resources such as social capital (Greenhaus and Powell, 2006), personal learning (Lankau and Scandura, 2002), and organizational identification (Dutton et al., 1994) may be possible mediators.

Last, we did not include moderators. Leadership research has often not considered followers’ individual differences despite prior research suggesting that follower characteristics represent a key contextual variable in influencing leader behavior (Liden and Antonakis, 2009). Furthermore, ten Brummelhuis and Bakker (2012) argue that personality presumably plays an important moderating role in spill over effects between work and family. Hence, it would be desirable in future research to explore the intersection between the self-concept of followers and the behavior of leaders in order to contribute to a meaningful augmentation of theory development on spiritual leadership, personality, and the work-family interface.

### Managerial Implications

Our study suggests that spiritual leadership plays a pivotal role in promoting followers’ WFF by acting as a supportive resource for followers, fostering job resources, and allowing job resources to strengthen their PsyCap. Therefore, our work advances the idea that it is important to develop and practice spiritual leadership in order to foster employee job resources, PsyCap, and WFF. As inner life practice has been shown to be a source for spiritual leadership development (Fry and Nisiewicz, 2013; Fry et al., 2016), leadership training needs to include inner life practices, which, such as spending time in nature, meditation, reading inspirational literature, yoga, observing religious traditions, and writing in a journal in leadership development (Fry, 2008). Coaching and mentoring should help leaders engage in self-reflection and mindfulness about who they are, what they are doing, and where they are going (Fry, 2008), aiding them in paying attention to their inner life as a human being regardless of their different religious beliefs (Fry, 2008; Jeon et al., 2013). Furthermore, organizations should advise managers to place more emphasis on vision, hope/faith, and altruistic love when interacting with employees to develop a spiritual leadership style.

## Conclusion

From a resource perspective, we have theoretically established and empirically tested a conceptual model linking spiritual leadership, job resources, PsyCap, and WFF. In doing so, this study not only broadens the understanding of spiritual leadership theory beyond the traditional boundaries of the workplace, but also adds to a growing body of literature on work-family interface, job resources, and PsyCap. Our findings also have important implications for organizations and managers that must employ their followers’ available resources effectively to manage organizational success, individual well-being, and personal growth. We hope our theoretical framework and the supportive results of this study will stimulate additional research in these important fields of spiritual leadership, job resources, PsyCap, and work-family interface to help meet the unprecedented challenges facing organizations now and in the future.

## Data Availability Statement

The raw data supporting the conclusions of this article will be made available by the authors, without undue reservation.

## Ethics Statement

This study was carried out in accordance with the recommendations of the Ethical Principles of Psychologists and Code of Conduct by the American Psychological Association’s (APA). The protocol was approved by the employee’s council of the participating organizations as well as the ethics committee of Shandong University. The participants provided their written informed consent to participate in this study.

## Author Contributions

PJ: has written the introduction section, the theoretical framework, hypotheses development of the manuscript, and acquisition of data. CL: has done the formal data analysis and written the research methodology section, interpretation part of the data analysis section, the implications, limitations, and conclusion sections. Both authors contributed to the article and approved the submitted version.

## Conflict of Interest

The authors declare that the research was conducted in the absence of any commercial or financial relationships that could be construed as a potential conflict of interest.

## References

[B1] Anderson M. H. Sun P. Y. (2017). Reviewing leadership styles: overlaps and the need for a new ‘full-range’ theory. *Int. J. Manage. Rev*. 19 76–96. 10.1111/ijmr.12082

[B2] Avey J. B. Avolio B. J. Luthans F. (2011). Experimentally analyzing the impact of leader positivity on follower positivity and performance. *Leadersh. Q*. 22 282–294. 10.1016/j.leaqua.2011.02.004

[B3] Bagger J. Li A. (2014). How does supervisory family support influence employees’ attitudes and behaviors? A social exchange perspective. *J. Manage.* 40 1123–1150. 10.1177/0149206311413922

[B4] Bakker A. B. Geurts S. A. E. (2004). Towards a dual-process model of work-home interference. *Work Occup.* 31 345–366. 10.1177/0730888404266349

[B5] Baykal E. Zehir C. (2018). Mediating effect of psychological capital on the relationship between spiritual leadership and performance. *E a M Ekonomie a Manage.* 21 124–140. 10.15240/tul/001/2018-3-008

[B6] Boyar S. L. Mosley D. C. (2007). The relationship between core self-evaluations and work and family satisfaction: The mediating role of work-family conflict and facilitation. *J. Vocat. Behav.* 71, 265–281. 10.1016/j.jvb.2007.06.001

[B7] Braun S. Nieberle K. W. A. M. (2017). Authentic leadership extends beyond work: a multilevel model of work-family conflict and enrichment. *Leadersh. Q*. 28 780–797. 10.1016/j.leaqua.2017.04.003

[B8] Breevaart K. Bakker A. B. Hetland J. Demerouti E. Olsen O. K. Espevik R. (2013). Daily transactional and transformational leadership and daily employee engagement. *J. Occup. Organ. Psychol*. 87 138–157. 10.1111/joop.12041

[B9] Brislin R. W. (1980). “Translations and content analysis of oral and written materials,” in *Handbook of Cross-Cultural Psychology*, eds Triandis H. C. Berry J. W. (Boston, MA: Allyn and Bacon), 389–444.

[B10] Brown M. E. Treviño L. K. Harrison D. A. (2005). Ethical leadership: a social learning perspective for construct development and testing. *Organ. Behav. Hum. Dec*. 97 117–134. 10.1016/j.obhdp.2005.03.002

[B11] Butts M. M. Casper W. J. Yang T. S. (2013). How important are work-family support policies ? A meta-analytic investigation of their effects on employee outcomes. *J. Appl. Psychol.* 98 1–25. 10.1037/a0030389 23106685

[B12] Byron K. (2005). A meta-analytic review of work-family conflict and its antecedents. *J. Vocat. Behav.* 67 169–198. 10.1016/j.jvb.2004.08.009

[B13] Chen C. Yang C. (2012). The impact of spiritual leadership on organizational citizenship behavior: a multi-sample analysis. *J. Bus. Ethics* 105 107–114. 10.1007/s10551-011-0953-3

[B14] Chen C. Y. Li C. I. (2013). Assessing the spiritual leadership effectiveness: the contribution of follower’s self-concept and preliminary tests for moderation of culture and managerial position. *Leadersh. Q*. 24 240–255. 10.1016/j.leaqua.2012.11.004

[B15] Chen C. Y. Yang C. Y. Chun-I L. I. (2012). Spiritual leadership, follower mediators, and organizational outcomes: Evidence from three industries across two major Chinese societies. *J. Appl. Soc. Psychol.* 42, 890–938. 10.1111/j.1559-1816.2011.00834.x

[B16] Chen S. Jiang W. Zhang G. Chu F. (2019). Spiritual leadership on proactive workplace behavior: the role of organizational identification and psychological safety. *Front. Psychol*. 10:1206. 10.3389/fpsyg.2019.01206 31191401 PMC6540823

[B17] Chen S. C. Chiang Y. H. Huang Y. J. (2017). Exploring the psychological mechanisms linking work-related factors with work-family conflict and work-family facilitation among Taiwanese nurses. *Int. J. Hum. Resour. Manage.* 28 1–22. 10.1080/09585192.2015.1118140

[B18] Choi Y. E. Cho E. N. Jung H. J. Sohn Y. W. (2018). Calling as a predictor of life satisfaction: the roles of psychological capital, work-family enrichment, and boundary management strategy. *J. Career Assess.* 26 567–582. 10.1177/1069072717723092

[B19] Dutton J. E. Dukerich J. M. Harquail C. V. (1994). Organizational images and member identification. *Admin. Sci. Q.* 39 239–263. 10.2307/2393235

[B20] Friedman S. D. Greenhaus J. H. (2000). *Allies or Enemies? What Happens When Business Professionals Confront Life Choices.* New York, NY: Oxford University Press.

[B21] Fry L. W. (2003). Toward a theory of spiritual leadership. *Leadersh. Q.* 14 693–727. 10.1016/j.leaqua.2003.09.001

[B22] Fry L. W. (2008). “Spiritual leadership: state-of-the-art and future directions for theory, research, and practice,” in *Spirituality in Business: Theory, Practice, and Future Directions*, eds Biberman J. Tischler L. (New York, NY: Palgrave), 10–124.

[B23] Fry L. W. Latham J. R. Clinebell S. K. Krahnke K. (2016). Spiritual leadership as a model for performance excellence: a study of Baldrige award recipients. J. Manag. Spiritual. Relig. 14, 22–26. 10.1080/14766086.2016.1202130

[B24] Fry L. W. Nisiewicz M. (2013). *Maximizing the Triple Bottom Line through Spiritual Leadership.* Stanford, CA: Stanford Business Books.

[B25] Fry L. W. Vitucci S. Cedillo M. (2005). Spiritual leadership and army transformation: theory, measurement, and establishing a baseline. *Leadersh. Q.* 16 835–862. 10.1016/j.leaqua.2005.07.012

[B26] Gooty J. Gavin M. Johnson P. D. Frazier M. L. Snow D. B. (2009). In the eyes of the beholder: transformational leadership, positive psychological capital, and performance. *J. Leadersh. Organ. Stud.* 15 353–367. 10.1177/1548051809332021

[B27] Greenhaus J. H. Powell G. N. (2006). When work and family are allies: a theory of work-family enrichment. *Acad. Manage. Rev.* 31 72–92. 10.5465/AMR.2006.19379625

[B28] Grzywacz J. G. Butler A. B. (2005). The Impact of job characteristics on work-to-family facilitation: testing a theory and distinguishing a construct. *J. Occup. Health Psychol.* 10 97–109. 10.1037/1076-8998.10.2.97 15826221

[B29] Hackman J. R. Oldham G. R. (1975). Development of the job diagnostic survey. *J. Appl. Psychol.* 60 159–170. 10.1037/h0076546

[B30] Hobfoll S. E. (2002). Social and psychological resources and adaptation. *Rev. Gen. Psychol.* 6 307–324. 10.1037/1089-2680.6.4.307

[B31] Hobfoll S. E. Halbesleben J. Neveu J. P. Westman M. (2018). Conservation of resources in the organizational context: the reality of resources and their consequences. *Annu. Rev. Organ. Psychol. Organ. Behav.* 5 103–128. 10.1146/annurev-orgpsych-032117-104640

[B32] Hu L. Bentler P. M. (1999). Cutoff criteria for fit indices in covariance structure analysis: conventional criteria versus new alternatives. *Struct. Equ. Modeling* 6 1–55. 10.1080/10705519909540118

[B33] Hunsaker W. D. (2016). Spiritual leadership and organizational citizenship behavior: relationship with Confucian values. *J. Manage. Spirit. Relig.* 13 206–225. 10.1080/14766086.2016.1159974

[B34] Jeon K. Passmore D. Lee C. Hunsaker W. (2013). Spiritual leadership: a validation study in a Korean context. *J. Manage. Spirit. Relig.* 10 342–357. 10.1080/14766086.2013.801026

[B35] Jiang Z. Jiang X. (2015). Core self-evaluation and life satisfaction: the person-environment fit perspective. *Pers. Indiv. Differ.* 75, 68–73. 10.1016/j.paid.2014.11.013

[B36] Kossek E. E. Pichler S. Bodner T. Hammer L. B. (2011). Workplace social support and work-family conflict: a meta analysis clarifying the influence of general and work-family specific supervisor and organizational support. *Pers. Psychol.* 64 289–313. 10.1111/j.1744-6570.2011.01211.x 21691415 PMC3116443

[B37] Krishnakumar S. Houghton J. D. Neck C. P. Ellison C. N. (2014). The “good” and the “bad” of spiritual leadership. *J. Manage. Spirit. Relig.* 12 1–21. 10.1080/14766086.2014.886518

[B38] Lankau M. J. Scandura T. A. (2002). An investigation of personal learning in mentoring relationships: content, antecedents, and consequences. *Acad. Manage. J.* 45 779–790. 10.2307/3069311

[B39] Li A. McCauley K. D. Shaffer J. A. (2017). The influence of leadership behavior on employee work-family outcomes: a review and research agenda. *Hum. Resour. Manage. Rev.* 27 458–472. 10.1016/j.hrmr.2017.02.003

[B40] Liden R. C. Antonakis J. (2009). Considering context in psychological leadership research. *Hum. Relat.* 62 1587–1605. 10.1177/0018726709346374

[B41] Luthans F. Avey J. B. Avolio B. J. Norman S. Combs G. (2006). Psychological capital development: toward a micro-intervention. *J. Organ. Behav.* 27 387–393. 10.1002/job.373

[B42] Luthans F. Avolio B. J. Avey J. B. Norman S. M. (2007). Positive psychological capital: measurement and relationship with performance and satisfaction. *Pers. Psychol.* 60 541–572. 10.1111/j.1744-6570.2007.00083.x

[B43] McNall L. A. Scott L. D. Nicklin J. M. (2015). Do positive affectivity and boundary preferences matter for work-family enrichment? A study of human service workers. *J. Occup. Health Psychol.* 20 93–104. 10.1037/a0038165 25347683

[B44] Munn S. L. (2013). Unveiling the work-life system: the influence of work-life balance on meaningful work. *Adv. Dev. Hum. Resour*. 15 401–417.

[B45] Muthén B. O. (2011). *Applications of Causally Defined Direct and Indirect Effects in Mediation Analysis using SEM in Mplus.* Available online at: http://citeseerx.ist.psu.edu/viewdoc/download;jsessionid=2FBF07DB4C44F4B546DCE0C3769D69E7 (accessed January 3, 2020).

[B46] Muthén L. K. Muthén B. O. (1998-2012). *Mplus User’s Guide*, 7th Edn. Los Angeles, CA: Muthén & Muthén.

[B47] Perry S. J. Witt L. A. Penney L. M. Atwater L. (2010). The downside of goal-focused leadership: the role of personality in subordinate exhaustion. *J. Appl. Psychol.* 95 1145–1153. 10.1037/a0020538 20718524

[B48] Podsakoff P. M. MacKenzie S. B. Lee J. Y. Podsakoff N. P. (2003). Common method biases in behavioral research: a critical review of the literature and recommended remedies. *J. Appl. Psychol.* 88 879–903. 10.1037/0021-9010.88.5.879 14516251

[B49] Pugh D. S. Hickson D. J. Hinings C. R. Turner C. (1968). Dimensions of organizational structure. *Admin. Sci. Q.* 13 65–105. 10.2307/2391262

[B50] Reave L. (2005). Spiritual values and practices related to leadership effectiveness. *Leadersh. Q.* 16 655–687. 10.1016/j.leaqua.2005.07.003

[B51] Shek D. T. L. (2006). Perceived parental behavioral control and psychological control in Chinese adolescents in Hong Kong. *Am. J. Fam. Ther.* 34 163–176. 10.1080/01926180500357891

[B52] Siu O. L. Bakker A. B. Brough P. Lu C. Q. Wang H. Kalliath T. (2015). A three-wave study of antecedents of work-family enrichment: the roles of social resources and affect. *Stress Health* 31 306–314. 10.1002/smi.2556 26468889

[B53] Smircich L. Morgan G. (1982). Leadership: the management of meaning. *J. Appl. Behav. Sci.* 18 257–273. 10.1177/002188638201800303 10260212

[B54] Spector R. G. (2007). John trounce. *Br. J. Clin. Pharmacol.* 64 116–117. 10.1111/j.1365-2125.2007.02978.x

[B55] ten Brummelhuis L. L. Bakker A. B. (2012). A resource perspective on the work-home interface: the work-home resources model. *Am. Psychol.* 67 545–556. 10.1037/a0027974 22506688

[B56] Van Steenbergen E. F. Ellemers N. Mooijaart A. (2007). How work and family can facilitate each other: distinct types of work-family facilitation and outcomes for women and men. *J. Occup. Health Psychol.* 12 279–300. 10.1037/1076-8998.12.3.279 17638494

[B57] Voydanoff P. (2004). The effects of work demands and resources on work-to-family conflict and facilitation. *J. Marriage Fam.* 66 398–412. 10.1111/j.1741-3737.2004.00028.x

[B58] Wang M. Guo T. Ni Y. Shang S. Tang Z. (2019). The effect of spiritual leadership on employee effectiveness: an intrinsic motivation perspective. *Front. Psychol*. 9:2627. 10.3389/fpsyg.2018.02627 30662419 PMC6328445

[B59] Wang Y. D. Hsieh H. H. (2014). Employees’ reactions to psychological contract breach: a moderated mediation analysis. *J. Vocat. Behav*. 85, 57–66. 10.1016/j.jvb.2014.04.003

[B60] Wayne J. H. Grzywacz J. G. Carlson D. S. Kacmar K. M. (2007). Work-to-family facilitation: a theoretical explanation and model of primary antecedents and consequences. *Hum. Resour. Manage. Rev.* 17 63–76. 10.1016/j.hrmr.2007.01.002

[B61] Williams L. J. Hartman N. Cavazotte F. (2010). Method variance and marker variables: a review and comprehensive CFA marker technique. *Organ. Res. Methods* 13 477–514. 10.1177/1094428110366036

[B62] Xanthopoulou D. Bakker A. B. Demerouti E. Schaufeli W. B. (2007). The role of personal resources in the job demands-resources model. *Int. J. Stress Manage.* 14 121–141. 10.1037/1072-5245.14.2.121

[B63] Yang F. Liu J. Wang Z. Zhang Y. (2019). Feeling energized: a multilevel model of spiritual leadership, leader integrity, relational energy, and job performance. *J. Bus. Ethics* 158 983–997. 10.1007/s10551-017-3713-1

[B64] Zhang H. Kwan H. K. Everett A. M. Jian Z. (2012). Servant leadership, organizational identification, and work-to-family enrichment: the moderating role of work climate for sharing family concerns. *Hum. Resour. Manage.* 51 747–768. 10.1002/hrm.21498

